# Selenium and at-risk pregnancy: challenges and controversies

**DOI:** 10.1186/s13044-020-00090-x

**Published:** 2020-10-01

**Authors:** Leonidas H. Duntas

**Affiliations:** Evgenideion Hospital, Unit of Endocrinology, Metabolism and Diabetes, Thyroid Section, University of Athens, 20 Papadiamantopoulou Str, 11528 Athens, Greece

**Keywords:** Selenium, Selenoproteins, Selenomethionine, Selenoprotein-P, Thyroid autoimmunity, Pregnancy, Miscarriage, Preeclampsia, Premature birth

## Abstract

Selenium (Se), an essential trace element, is inserted as selenocysteine into an array of functional proteins and forms the core of various enzymes that play a cardinal role in antioxidant defense mechanisms, in redox regulation, and in thyroid hormone metabolism. Variations in plasma Se are due to nutritional habits, geographic and ethnic differences, and probably to genetic polymorphisms, the latter still to be conclusively established. Se concentrations were reported to be low in women of reproductive age in the UK, decreasing further during pregnancy, this resulting in low plasma and placental antioxidant enzyme activities. Since low serum Se levels have been found in women with preeclampsia, it has been hypothesized that low maternal Se status during early gestation may be an indicator of preterm birth. Moreover, it is documented that Se administration during pregnancy tendentially reduced the markers of thyroid autoimmunity and the incidence of maternal hypothyroidism in the postpartum period. Importantly, low Se levels in pregnant women affect fetal growth and augment the risk of delivering a small-for-gestational age infant by reducing placental antioxidant defense, while low Se in the third trimester is thought to indicate increased demands by the placenta, an issue which requires further confirmation. There is evidently a need for double-blind, placebo-controlled studies to better determine the efficacy and safety of Se supplementation in pregnancy at high risk for complications, and for measurement of Se levels or of selenoprotein P, the most reliable parameter of Se status, particularly in selenopenic regions.

## Introduction

**Fig. 1:**
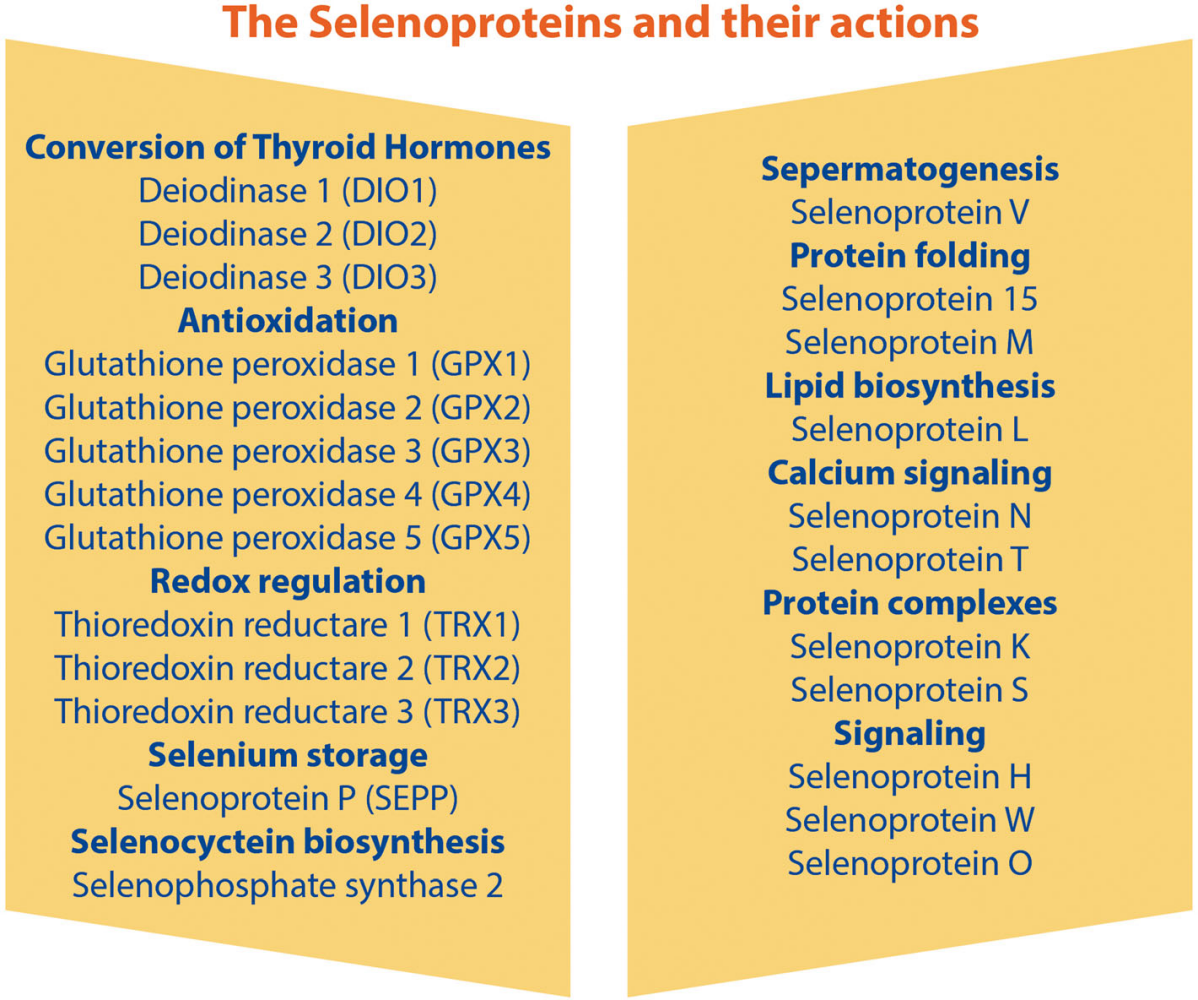
Selenoproteins and their actions

Selenium (Se), in the form of the 21st proteinogenic amino acid selenocysteine (SeCyS), is incorporated in specific proteins called selenoproteins, the most important being the deiodinases (DIOs), gluthathione peroxidases (GPXs), thioredoxin reductases (TRXR), SeCys insertion binding protein 2 (SECISBP2), and the Se transport protein (SELENOP) [1, 2]. DIOs, by regulating the conversion of thyroxine (T4) to triiodothyronine (T3) and reversing triiodothyronine (rT3) and thyroidonamines, control thyroid hormone turnover [3]. Meanwhile, Se-dependent GPXs and TRXR are implicated in thyroid gland protection through modulating redox activities [4] **(**Fig. 1**).**

Se deficiency has been associated with autoimmune thyroid diseases (AITD) and sepsis, arteriosclerosis, cardiovascular disease, cancer, increased mortality among the elderly and hemodialysis patients, and cognitive decline [5, 6]. Importantly, low Se status has been associated with adverse pregnancy outcomes such as miscarriages, neural tube defects, premature birth, low birth weight, preeclampsia, glucose intolerance, gestational diabetes, and even diaphragmatic hernia [7–10], albeit its precise role in the etiology of these complications has yet to be clarified. It is also noteworthy that Se deficiency has been related to adverse outcomes in human immune deficiency virus (HIV)-infected pregnant women and their offspring [11]. Lastly, women with Se deficiency, when compared to women with normal Se concentrations, showed an approximately eight-fold higher risk of preterm delivery) and of delivering at term a low birth weight infant [12]. However, although the effect of Se supplementation on pregnancy outcome appears promising, there are as yet no firm recommendations for its implementation [13].

The relationships between first trimester levels of Se, iron (Fe), zinc (Zn), and copper (Cu) and pregnancy outcome were recently studied in a Polish prospective cohort of 563 women [14]. An increase in Se levels by 1 μg/l was observed to reduce the risk of gestational hypertension in a multivariate logistic regression by 6% (; *p* = 0.004), the risk of intrauterine growth retardation (IUGR) by 11% (*p* = 0.013), and the risk of preterm birth by 7% (; *p* = 0.061).

This review aims to summarize the current knowledge regarding Se deficiency and excess in pregnancy outcome as well as to briefly describe the challenges and controversies concerning Se supplementation in pregnant women with Se deficiency with or without concomitant thyroid disease.

### The biochemistry and bioavailability of se metabolism

**Fig. 2:**
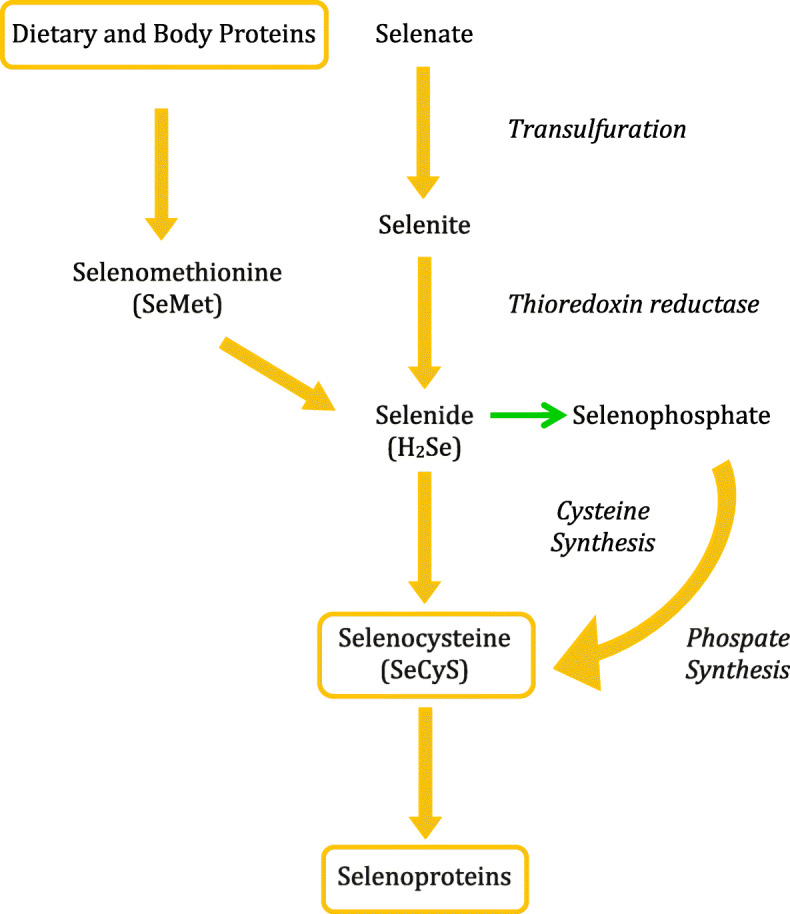
The main metabolic steps of metabolism of inorganic and organic Se Both inorganic (selenite) and organic (selenomethionine, SeMet) are converted to selenide (H_2_ Se) before the formation of selenocysteine (SeCys) and its insertion in bioactive selenoproteins.

Dietary Se is mainly obtained from cereals, bread, nuts, fish, poultry, and meat, while remarkable variations in plasma Se exist depending upon location and soil content [15]. Se is ingested as inorganic selenides or selenates, the main Se source in soils, this being reduced to selenite which is metabolized to selenide (H_2_Se) via selenodiglutathione and glutathione selenopersulfide. Alternatively, it may be directly reduced by TRXR to H2Se, which serves as substrate for the utilization and excretion of Se and the biosynthesis of SeCys [16, 17]. H2Se can also form, by cysteine synthase, SeCyS, which is cotranslationally inserted into selenoproteins, while it can also synthesize selenophosphate, which is the link to selenoprotein synthesis by selenophosphate synthetase **(**Fig. 2**)**. Interestingly, SeCyS is efficiently methylated by certain plants, such as *Brassica oleracea* capitata (kale), *Brassica juncea*, and *Allium tricoccum*, which can accumulate high concentrations of Se in the form of selenomethionine (SeMet), resulting in increased levels of methylated metabolites [17, 18]. It is also important to note that both inorganic and organic forms must first be converted to inorganic H_2_Se before the synthesis of SeCyS, which crucially contributes to the formation of selenoproteins **(**Fig. 2**).** Following absorption of SeMet from the intestinal tract, the amino acid can be found incorporated into proteins in place of methionine through acylation of methionine-translation ribonucleic acid (Met-tRNA) [16]. SeMet is absorbed and retained more efficiently than the inorganic forms, while prolonged SeMet administration, dependent on the dose, increases (baseline: 76.5 ± 2.47 vs. 82.8 ± 3.28 μg/l following ingestion of 166 μg SeMet/14 days) circulating blood Se concentration measured by hydride generation atomic fluorescence spectroscopy [19]. Chronic high intake of organic Se may result in elevated levels of SeMet and in its cleavage product, methyl selenol, which can disrupt redox-regulated cell signaling and, in the form of selenolates (RSeˉ), induce oxidative stress by generating superoxide radicals [20, 21]. Signs of toxicity, such as alopecia and dermatitis, have been seen following an intake of 300 μg/day over a long period and may result in unexpectedly increased mortality after 5 years [21]. If supplementation is deemed advisable due to inadequate Se intake, organic Se in the form of SeMet is preferable to inorganic Se, as the former binds to proteins and is retained more efficiently [22]. Due to its progressive increase, periodic monitoring of serum Se is recommended. Pregnant women excrete less urinary Se than nonpregnant and the amount progressively diminishes as pregnancy advances, possibly to conserve the trace element for the increasing fetal and maternal demands of pregnancy [23].

### Se and impact on the fetus and child

In order to ensure well-functioning metabolism, it is vital to have a balanced diet including a sufficiency of macronutrients and micronutrients—this being, of course, equally essential for a healthy pregnancy. There is some evidence in the literature suggesting that suboptimal Se intake during pregnancy is a common phenomenon [24] and that inadequate micronutrient intake during this period may result in poor fetal growth and development as well as poor pregnancy outcome [25]. For instance, decreased serum Se and iron have been associated with pregnancy complications, although there is as yet scant information regarding the specific role of Se during pregnancy [26]. This may be due to various study limitations, such as variability of the study populations, non-homogeneous groups, unspecified sample size, different analytical methods employed, and transportation of selenium to the fetus: thus, conclusions about causal relationships cannot be drawn [27].

Se plays a multivariable role during pregnancy. Though to date there is a paucity of studies evaluating its benefits v. toxicity during this period, a possible link between Se and neurodevelopment in early life has been proposed.

Recently, the association between maternal Se levels and neuropsychological developments were analyzed in 650 mother-child pairs from the Spanish Childhood Environmental Project [28], and mean serum Se levels measured during the first trimester were observed to be 79.7 μg/L. However, the multivariate analysis did not find any significant inverse linear relationship between Se concentrations and standardized mental and psychomotor development scores. Importantly, among children with the AG + AA genotype for rs6970396 Se metabolizing indolethylamine N-methyltransferase gene (INMT), an inverted U-shaped association between Se and neuropsychological development was identified, albeit a descending curve was indicated for the GG genotype [28]. It is of note that during the prenatal period, a complex balancing act occurs between Se toxicity and benefits, which should be particularly taken into account during pregnancy.

In a recent population-study including 539 mother-child pairs from the Polish Mother and Child Cohort, Se levels were quantified in each trimester of pregnancy as well as at delivery and in cord blood, while psychomotor development was assessed in children at the age of 1 and 2 y [29]. Plasma Se levels decreased through the entire pregnancy from 48.3 ± 10.6 μg/l in the first trimester to 38.4 ± 11.8 μg/l at delivery. A statistically significant positive association was observed between Se levels in the first trimester of pregnancy and motor development at 1 y of age, as well as language development at 2 y of age [29]. These results indicate an association between Se levels in the first trimester and motor neurodevelopment, although no causality has so far been deduced. It should be emphasized that the very low Se levels observed during the entire pregnancy were the determining factor in the resulting differences between the Polish study and the abovementioned Spanish study.

In a recent Croatian study aiming to evaluate the association of maternal and cord blood Se levels with neonatal cerebellum measures and child neurodevelopment at the age of 18 months, 205 mother-child pairs from the Croatian Mother and Child Cohort were investigated [30]. Mean Se levels in maternal blood and cord blood were 92.6 ng/g and 97.0 ng/g, respectively. While a moderate negative correlation (*r* = − 0.372; *p* = 0.008) between Se levels and cerebellum length was observed, in the group of female children, cord blood Se levels were positively correlated with cerebellum width, measured via cranial ultrasonography. Furthermore, there was a weak but positive correlation (*r* = 0.176; *p* = 0.029) between maternal blood Se levels and the children’s cognitive abilities. This study therefore indicated that prenatal Se intake correlates with cerebellum length and width, suggesting that the cerebellum may be used as a potential biomarker for detection of possible adverse effects due to insufficiency of micronutrients, particularly that of Se.

In contrast, in a combined study in Boston and New York recruiting 1068 pairs with maternal fish intake of 1.7 servings/week and showing median Se levels of 205.6 ng/ml, there was no evidence of an association of maternal prenatal fish intake, or of mercury (Hg), or of Se status with verbal or non-verbal intelligence, visual motor function, or visual memory at median 7.7 y of age [31]. These results were obtained by multivariable linear regression analyses, adjusting for maternal and child characteristics, including home environment and maternal intelligence.

However, it should be mentioned that in this US study, prenatal Se levels were much higher than those reported in European studies, which presented a large disparity, ranging from very low levels in Poland to borderline low levels in Spain and normal Se levels in Croatia. The implications, however, are that low maternal blood Se levels are positively correlated with the offspring’s cognitive abilities, while an increase in maternal Se concentration is followed by improvement in children’s cognitive and motor scores. On the other hand, maternal blood Se levels were negatively correlated with cerebellum length and Se intake. These results need confirmation regarding gender difference of the offspring, while geographical comparison should be conducted of prenatal Se levels as reported in various studies and their respective effects on neurodevelopment and children’s cognitive abilities.

### Se and trophoblast physiology

Trophoblasts, cells forming the outer layer of the blastocyst, contribute to implantation, placenta development, and oxygen and metabolite exchange between the embryo and the mother, leading to successful fetal development [32]. Therefore, well-functioning trophoblasts are indispensable for an uncomplicated pregnancy. It is of particular note that trophoblasts begin to invade the endometrium (endoglandular trophoblast) already at the time of implantation, enabling nutrition of the embryo before the perfusion of the placenta with maternal blood [33]. There follows invasion of endovenous trophoblasts into the uterine veins, this enabling, throughout pregnancy, drainage of fluids originating in the placenta back into the maternal circulation. At this point, endoarterial trophoblasts invade the spiral arteries, which permits, already from the second trimester of pregnancy, hemotrophic nutrition of the fetus [34]. Armed with the above data, which have recently come to light, we are now in a position to identify alterations possibly taking place in pathological pregnancies, ranging from tubal pregnancies to recurrent spontaneous abortions.

Se sufficiency is likely to play a fundamental role in this natural process. A 2017 study showed that knockdown of SECISBP2 suppressed the migratory and invasive abilities of trophoblast while reducing the levels of some selenoproteins, including DIO2, GPX1, and selenoprotein K (SELENOK), and increasing those of malondialdehyde, an index of oxidative stress (OS) [35]. This effect, mediated by inactivation of the phosphoinositide 3-kinase activation (PI3K/AKT) and extracellular-signal-regulated kinase (ERK) signaling pathways, offers an insight into the complex mechanisms of miscarriage and preeclampsia during Se deficiency.

**Fig. 3:**
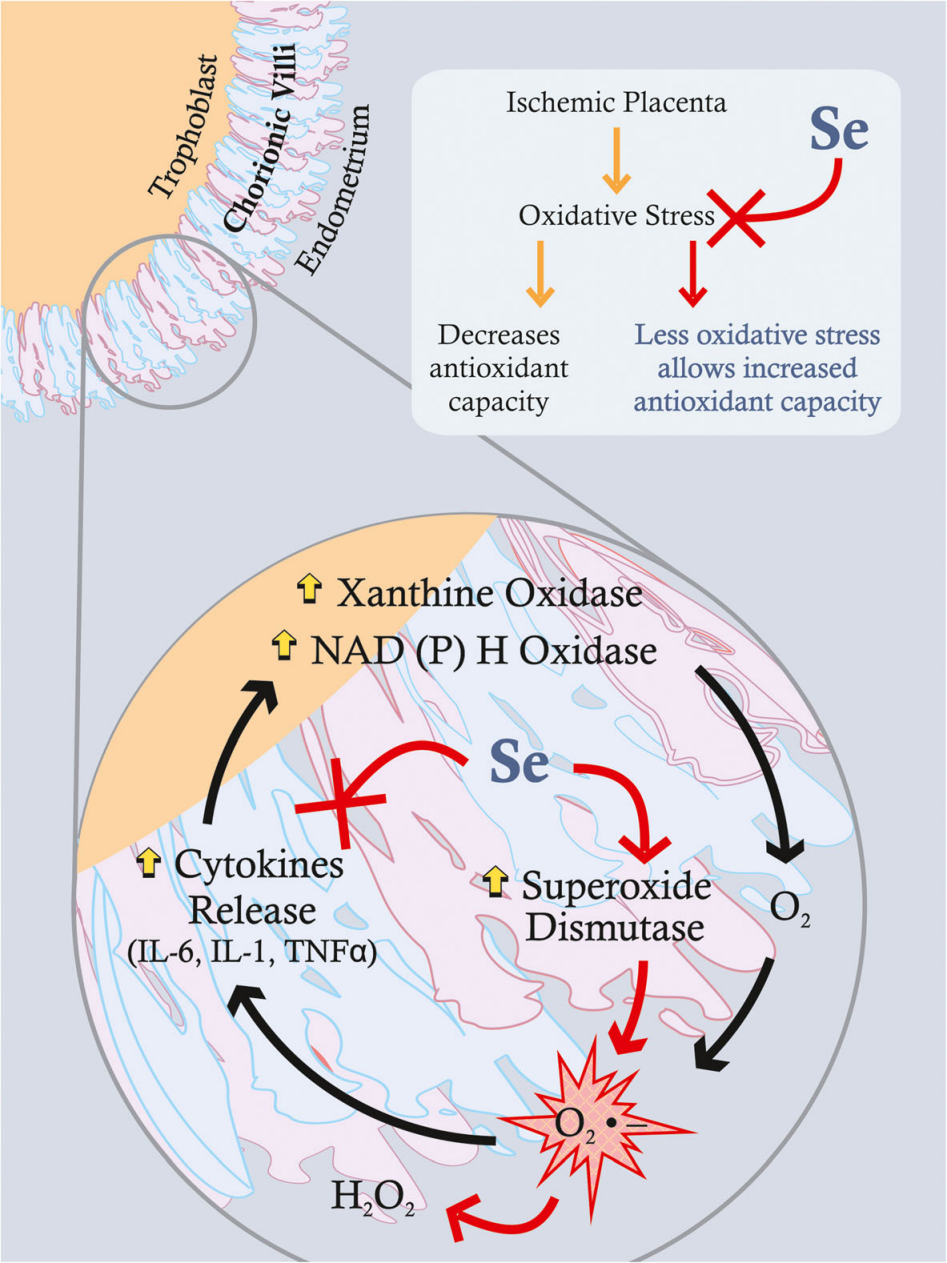
Uterine arterial hypoxia, or placental ischemia, triggers oxidative stress (OS), generating superoxide radical (O_2_^*^) as a byproduct by specific enzymes such as xanthine or nicotinamide adenine dinucleotide phosphate (NADPH) oxidases. O_2_^*^ may stimulate the production of cytokines (particularly IL-6), thus increasing the intensity of OS and inflammation and further increasing the generation of O_2_ *_,_ leading to such adverse pregnancy outcomes as altered placental growth, reduced fetoplacental blood flow, and preeclampsia. Sufficient availability of Se may alleviate OS by increasing antioxidant capacities, such as superoxide dismutase (SOD) which converts O_2_ * to hydrogen peroxide (H2O2), and inhibiting cytokine formation and activity

Trophoblasts, in the process of connecting with maternal blood vessels, accelerate villi formation while expanding their surface area, which leads to increased intervillous space perfusion: OS levels are thus increased, triggering the expression of heat shock protein (hsp60 and hsp70), which results in retardation of fetal development and possible pregnancy failure [36]. OS, defining an imbalance between free radical oxygen species (ROS) generation and antioxidant defense, is considered as a key factor in the pathogenesis of adverse pregnancy outcomes [37]. An antioxidant reaction to OS occurs through upregulation of the expression of such genes as superoxide dismutase and heme oxygenase 1, this heightening mitochondrial activity [38] **(**Fig. 3**).** Recently it was reported that Se supplementation induced mitochondrial biogenesis in trophoblastic cancer cell lines by increasing the expression of selenoprotein H, and that Se-treated trophoblasts, under hypoxic conditions, increased mitochondrial membrane potential while decreasing ROS levels [39]. Thus, by regulating mitochondrial activity, Se has the potential to improve the survival rate and invasive ability of trophoblasts, though the mechanisms of the trophoblast response to OS are not so far completely understood.

Se, being a cofactor of GPX which protects body cells from damage by free radicals, is essential for reduction of inflammation and maintenance of the integrity of cell function, while Se deficiency predisposes to inflammation and diminishes the inflammatory response: supplementation effectively attenuates inflammatory activity [40].

### Se and miscarriage

Se deficiency has often been observed in the setting of adverse pregnancy outcome, including miscarriage, preeclampsia, and preterm birth [41]. Specifically, when OS, which is generated during normal placental development, is exaggerated because of heightened ROS production, but also if there is reduced presence of antioxidant micronutrients in the placenta and maternal circulation, adverse pregnancy outcomes may arise [41]. In 2001, a pioneering study in this field conducted in Wales, including 40 women with first trimester nonrecurrent miscarriage, measured serum Se, albumin, and total protein concentration [42]. The results were compared with 40 nonpregnant, age-matched, healthy volunteers, and also with those of 40 pregnant women attending the antenatal clinic for booking in the first trimester. A reduction in serum Se occurred in the first trimester of pregnancies that progressed to term. However, a further highly statistically significant decrease (, *P* < 0.0001) in serum Se was observed in women who miscarried, clearly indicating a biological link of low Se levels with miscarriage [42]. The authors have also hypothesized a contribution of deoxyribonucleic acid (DNA) damage, i.e., destruction and fragmentation of DNA bases, as a result of the low antioxidant capacities attributed to Se deficiency and concomitantly high levels of ROS.

In a 2001 study from Poland, Se concentrations were measured in whole blood and plasma of women who had had a miscarriage: while levels were similar to those of women during the same period with viable pregnancy, they were significantly lower compared with healthy, age-matched, non-pregnant controls [43]. Glutathione levels were significantly higher in women with miscarriage as compared with viable pregnancies and with non-pregnant women. Red cell and plasma glutathione peroxidase activity of women who had had a miscarriage were significantly lower than in normal and control women [43]. In a more recent study from Indonesia, in 46 patients with spontaneous abortions, serum Se levels were significantly lower than those of women with normal pregnancies; however, glutathione peroxidase activity was similar in both groups. In a logistic regression analysis separating subjects into smoking and nonsmoking groups, total serum Se concentration, but interestingly, not serum glutathione peroxidase activity or smoking, were significantly correlated with the incidence of miscarriage [44]. Although most studies have reported low Se levels in women following miscarriages, there are some disparities, as others did not find any difference in hair Se content between pregnant women who miscarried and controls [45].

Despite the fact that a causal effect has not to date been demonstrated and large randomized control studies are still lacking, Se supplementation during the first trimester, particularly in those women who reside in low-Se areas and/or who are at high risk for miscarriage should be considered and administered on an individual basis, while quantification at the beginning of pregnancy is recommended in the above cases.

### Se and preeclampsia

Hypertensive pathologies often occur in pregnancy, particularly during the late second and the third trimesters with Se deficiency appearing to be strongly implicated [46]. Preeclampsia is a multisystem disorder that can have disruptive effects on the mother and the fetus and is major cause of death among pregnant women. It has been estimated that about 15% of premature births are attributable to preeclampsia [47]. OS is widely implicated as a major pathogenetic factor resulting from reduced antioxidant defense pathways, particularly involving GPX which is potentially linked to decreased Se availability [48]. Reduced GPX could trigger increased generation of toxic lipid peroxides contributing to the endothelial dysfunction and hypertension of preeclampsia. Though in the 2000s early supplementation with antioxidant vitamins C and E in women at risk of preeclampsia was recommended to reduce the risk and perinatal complications, randomized controlled trials since then have shown little to no benefit of this practice [49]. Whether this indicates that an inappropriate antioxidant strategy was used or that supplementation was administered too late in gestation to be beneficial, or else that there is de facto no effect, is not at present known.

However, in 2014, a double-blind, placebo-controlled trial was conducted with 230 primiparous pregnant women randomized to Se (60 μg/d, in form of yeast) or placebo treatment from 12 to 14 weeks of gestation until delivery [8]. The aim was to investigate whether a small increase in the Se intake of pregnant women with low Se status would protect against the risk of preeclampsia, as assessed by preeclampsia biomarkers. Whole-blood Se concentration was measured at baseline and at 35 weeks while plasma SELENOP concentration was determined only at 35 weeks. Significantly higher concentrations of whole-blood Se and plasma SELENOP were observed in the Se-treated group at 35 weeks than in the placebo group in which both parameters continuously decreased significantly. Of note, serum soluble vascular endothelial growth factor receptor-1 (sFlt1), an antiangiogenic factor linked with the risk of preeclampsia, was decreased in the Se treated group by week 35, particularly in those with the lowest quartile of Se status at baseline as compared to the placebo group [8]. Thus, Se supplementation may potentially reduce the risk of preeclampsia in pregnant women with low Se levels; however, these results need to be confirmed in large studies.

In a comprehensive 2018 study, the levels and activities of several key antioxidants and oxidant/pro-oxidants were investigated in Saudi patients with recurrent preeclampsia (RP) [50]. The levels of enzymatic antioxidants GPX, GSR, SOD, and CAT and non-enzymatic antioxidant micronutrient levels (Se, Zn, manganese) were considerably decreased, while significant increases of OS markers were observed in the plasma of RP patients in relation to those of healthy pregnant women [50]. The data indicated a shift in favor of OS in placental tissue of preeclamptic patients compared to healthy pregnant/non-pregnant individuals, and may imply that administration of Se and Zn may counterbalance the impact of OS.

A large Norwegian case-control cohort genetic analysis was undertaken to investigate potential associations between the G-105A promoter polymorphism of the inflammatory mediator selenoprotein S (SELENOS1) and preeclampsia [51]. It was reported that women with preeclampsia were 1.34 times more likely to have the GA or AA genotype and 1.22 times more likely to carry the A allele. Therefore, the A allele of the SELENOS1-105G > A polymorphism was postulated as a significant risk factor for preeclampsia [51].

Of special note was an analysis of maternal micronutrient concentrations together with associated antioxidant enzymes and SNPs in the subjects’ encoding genes in a prospective cohort study recruiting women at 15 weeks of pregnancy, who later developed preeclampsia [52]. No association was detected between the genotype for SNPs and antioxidant enzyme activity, although copper and ceruloplasmin were seen to be slightly increased among the women who developed preeclampsia. The authors concluded that the small elevation in copper could have contributed to OS at a later stage of pregnancy. However, since the hypothesis that functional SNPs might affect antioxidant enzyme activity in pregnant women has not to date been supported by solid evidence, a role for these genes in the etiology of preeclampsia does not appear plausible. On the other hand, in another study from the UK, it was shown that the genetic variant rs921943 in the dimethylglycine dehydrogenase (DMGDH) gene is significantly associated with Se status in UK pregnant women [53]. More specifically, women who carry the SELENOP1 rs3877899 A allele maintain their Se status much better during pregnancy, while their GPX3 activity further increases with supplementation, which suggests that they cope more adequately with low Se status [53].

Se was also negatively correlated with Cu in women with second-trimester induced abortion resulting from neural tube defects [54]. By depleting Se, Cu may increase OS and, hence, the risk for preeclampsia. It is therefore conceivable that the induction and consequences of OS are due not merely to a single mechanism, but that an interaction among trace elements and vitamins together with ethnic and genetic factors are involved.

Furthermore, in another recent study, it was demonstrated that Se levels in the lowest quartile (≤58 μg/L), quantified in weeks 10–14 in 121 hypertensive pregnant women, might be considered as a high-risk factor (*p* = 0.002) for pregnancy-induced hypertension, as compared to the highest quartile (> 67 μg/L) [55]. The risk was calculated using multivariate logistic regression analysis and after adjusted for confounders such as high BMI and smoking.

### Se and impact on preterm birth

Preterm birth, which occurs in 5–13% of pregnancies and is a major cause of perinatal morbidity and mortality, has been associated with Se deficiency. A study was conducted with 1197 white Dutch primiparous women, who were followed prospectively from 12 weeks of pregnancy to delivery with Se measurements [56]. A total of 60 women (5.3%) had a preterm birth and 21 had premature rupture of the membranes, while 13 had preeclampsia. Serum Se concentrations at 12 weeks’ gestation were observed to be considerably lower among women with preterm birth than among those who delivered at term. At 12 weeks’ gestation, the women were grouped by quartile of serum Se concentrations. Women in the lowest quartile of serum Se had double the risk of preterm delivery as women in the upper three quartiles. However, low serum Se levels at the end of the first trimester, while being associated with preterm delivery, were independent of occurrence of preeclampsia [56]. The latter findings reinforce the etiological relationship between low Se concentrations and pregnancy adverse outcome, especially during the first trimester, with high risk for both preterm birth and preeclampsia.

A nested case-control investigation recently conducted in Malawi included 181 women, 90/181 (49.7%) term and 91/181 (50.3%) preterm pregnant women [57]. Se levels were analyzed together with Zn and Cu. The overall mean serum Se level was 77.0, of Cu it was 2.50, (); and of Zn it was 0.77, (), with reference values of 47–142 μg/L, 0.76–1.59 mg/L, and 0.59–1.11 mg/L, respectively; no difference between Se and Zn was observed between term and preterm births. These findings may suggest absence of any Se deficiency but presence of high serum concentrations of Cu and Zn, these possibly accounting for the cases of preterm birth. The possible causes of the deficiencies may be geographical differences in microelements during pregnancy and/or ethnicity.

In a prospective birth cohort study conducted in Shanghai, 1931 women between 28 and 36 weeks of pregnancy were investigated [58]. Maternal serum Se levels < 103.7 μg/L (P25th) were significantly associated with a decrease of 0.014 μIU/mL in TSH levels while Se levels ≥103.7 μg/L were not significantly associated with TSH The fact that maternal TSH values were significantly negatively associated with infant birth weight implies that low Se status during pregnancy may be related to low birth weight and low thyroid function.

In a recent prospective cohort study, 750 women were recruited during the 10th–14th week of a singleton pregnancy aiming to investigate whether maternal serum Se in early pregnancy may be a risk marker for small-for-gestational age (SGA) birth weight [59]. Se concentrations were lower in mothers in the SGA group compared to the appropriate-for-gestational age infants group (59.60 vs. 62.54 μg/L, *p* = 0.020). Meanwhile, women in the lowest quartile of Se (≤56.60 μg/L) had an approximately three-fold higher risk of SGA compared to women in the higher quartiles (OR = 3.02; *p* = 0.019). Therefore, although the results need confirmation, this study indicates that maternal serum Se levels in early pregnancy can be a risk marker of SGA newborns.

In this line of evidence, an experimental study using an established mouse model, demonstrated that moderate Se deficiency reduced messenger RNA (mRNA) expression of selenoprotein N (SELENON), a selenoprotein that plays an important role in early embryogenesis, and of SELENOP [60]. It is of particular interest that DIO1 did not change, whereas DIO2 and DIO3 were decreased. It may therefore be hypothesized that the unchanged DIO1 increases T3 in maternal circulation, while reduced DIO2 and DIO3 levels in the placenta may not sustain adequate T4 and T3 concentrations, possibly resulting in delayed growth, thyroid hormones being highly involved in nutrient transporter activity. Se deficiency may thus affect fetal growth by dysregulating placental nutrient transport [60].

### Se and thyroid autoimmunity during pregnancy

The three steps of thyroid hormone (TH) synthesis, i.e. oxidation of iodide, iodination of tyrosine residues of thyroglobulin (Tg), and the oxidative coupling of iodinated tyrosine residues to generate Tg-bound TH, are catalyzed by the enzyme thyroid peroxidase (TPO), which is located in the apical membrane of the thyrocyte generating hydrogen peroxide (H2O2) as a substrate [61, 62]. H2O2 is produced in high concentrations by the nicotinamide adenine dinucleotide phosphate (NADPH)-dependent flavoproteins thyroid oxidase DUOX (dual oxidases) 1 and 2 and provides oxidative equivalents for TPO activity [63]. The thyroid gland is constantly exposed to variable concentrations of H2O2-derived ROS, which most likely arise as by-products of the above processes. H2O2 can readily disperse into the cytoplasm and nucleus, resulting in abnormal oxidation and iodination of proteins and lipids while also setting off apoptosis and inducing DNA damage [63].

In this context, according to an Italian study, Se administration during pregnancy in patients with autoimmune thyroiditis (AIT) results in a significant decrease of thyroid peroxidase antibodies (TPOAb) and the occurrence of postpartum (PP) hypothyroidism [64]. However, Se supplementation was not observed to exert a stronger effect than placebo in reducing TPOAb concentrations or the prevalence of TPOAb positivity during the course of pregnancy. In women who were TPOAb positive at baseline, a tendency towards reduction in TSH and FT4 levels was noted, while, at 35 weeks, the concentrations were lower than those of the placebo group (*P* = 0.050 and *P* = 0.029, respectively).

The SERENA study, a multicenter, randomized, double-blind, placebo-controlled trial, organized and promoted by the Young Italian Endocrinologists Group (EnGioI)—Italian Society of Endocrinology, aimed to evaluate the effect of Se supplementation on AIT during and after pregnancy [65]. A significant reduction of TPOAb and thyroglobulin antibodies (TGAb) postpartum titer was observed in patients treated with SeMet (83 μg/day) [at PP: TGAb 20 IU/ml (11.59–52.60), *p* < 0.01; TPOAb 255.00 IU/ml (79.00–292.00), *p* < 0.01], while antibody titer rebound was reported in the placebo group. These results confirmed the study by R. Negro [64], demonstrating that Se supplementation at a dosage of 83mcg/day during pregnancy and PP is safe and effective in decreasing autoantibody titers, while no effect on TH was found [Table 1].

**Table 1:** Observational and double-blind, placebo-controlled studies showing the impact of Se on fetus, preeclampsia and thyroid autoimmunity during pregnancy

Author	No of participants	Study design	Outcome	Ref.
***Se Impact on fetus***				
Polanska K 2017	539 mother-child pairs	Observational	1st trimester Se status was associated with child language and motor skills (β = 0.18, *p* = 0.03 and β = 0.25, *p* = 0.005, respectively) at one year of age	[24]
Mocenic I 2019	205 mother-child pairs	Observational	Maternal blood Se was negatively associated with cerebellum length (*r* = − 0.372; *p* = 0.008); cord blood was positively associated with cerebellum width (*r* = 0.613; *p* = 0.007)	[30]
Amoros R 2018	650 mother-child pairs	Observational	inverted U-shaped relationships between Se concentrations and mental and psychomotor development scores (β (95% CI) = − 0.13 (− 0.29, 0.03) and β (95% CI) = − 0.08 (− 0.24, 0.07), respectively)	[28]
***Se and preeclampsia***				
Rayman MP 2014	230 pregnant women	Double-blind placebo-controlled. Se 60 μg/day placebo. 12–14 week until delivery.	Soluble vascular endothelial growth factor receptor-1 (sFlt-1) decreased as marker of risk of preeclampsia	[8]
***Se and autoimmunity in pregnancy***				
Mao JJ 2016	230 pregnant women	Double-blind placebo-controlled. Se 200 μg/day or placebo until delivery.	In ThyAB+ TSH decreased (*p* = 0.050) vs. placebo. TSH in ThyAB- increased and FT4 decreased significantly during gestation (*p* < 0.001).	[67]
Negro R 2007	2143 euthyroid pregnant women	Prospective, randomized, placebo-controlled	Post-partum and permanent hypothyroidism were lower in women treated with SeMet 200 μg/day (28.6 vs. 48.6%, *p* < 0.01; and 11.7 vs. 20.3%, *p* < 0.01, respectively)	[64]
Mantovani G 2019	45 pregnant women (SERENA study)	multicenter, randomized, double-blind, placebo-controlled	Beneficial effect of SeMet treatment on ThyAB titer as compared to placebo (TgAb 151.03 ± 182.9, *p* < 0.01; TPOAb 441.28 ± 512.18, *p* < 0.01) and PP TgAb 19.86 (11.59–52.60), *p* < 0.01; TPOAb 255.00 (79.00–292.00), *p* < 0.01	[65]

In a region of low Se intake in Poland, serum Se and SELENOP concentrations were observed to be low in both healthy subjects (HS) (*n* = 45) and women with AIT (*n* = 29) [66]. From the first to third trimester, TPOAb and TGAb declined in AIT subjects by 71 and 60%, respectively. The decline in TPOAb and TGAb was unrelated to Se status. Serum Se concentrations showed only a slight positive correlation to TPOAb. In the AITD group, there was a statistically significant decline in serum Se concentrations from the 1st to 3rd (*p* < 0.001) and 2nd to 3rd trimesters (*p* < 0.01). In the HS group, mean Se concentrations declined with statistically significant differences between the 1st and 2nd (*p* < 0.01), the 1st and 3rd (*p* < 0.001), and, moreover, the 2nd and 3rd trimesters (*p* = 0.004) [66]. SELENOP concentrations declined concomitantly, reaching a level of statistical significance (*p* < 0.005). A positive correlation of serum Se and SELENOP was observed throughout the three trimesters in the entire group of pregnant women (*r* = 0.55; *P* < 0.001 in the first trimester; *r* = 0.34; *p* < 0.01 in the second trimester; and *r* = 0.51; *p* < 0.001 in the third trimester), suggesting that the average Se intake was below the level required for activation of SELENOP.

Therefore, the substantial Se deficiency which developed independent of AIT status in a large proportion of pregnant women (28.6% in AIT vs. 35.5% in HS) may be regarded as a preventable risk factor for complications during pregnancy.

A secondary analysis of Se in the double-blind, randomized, placebo-controlled Se in PRegnancy INTervention (SPRINT) study that enlisted 230 women with singleton pregnancies at 12 weeks of gestation [67] is of particular interest. The women were randomized to low Se (60 μg/day) or placebo until delivery. No statistically significant change in TPOAB or TgAB titers was shown, while TSH levels increased and FT4 decreased in women with negative thyroid antibodies throughout gestation (*P* < 0.001). In women with positive TPOAB and/or TgAB, TSH decreased and was lower than that of the placebo group at delivery (*p* = 0.050). The results suggest that low-dose Se administration in women with low-to-moderate iodine deficiency has no effect on autoimmune thyroid parameters, but it tendentially improves thyroid function in these women.

In summary, Se deficiency during pregnancy has been associated with a range of adverse effects on the fetus and mother and increased biomarkers of OS. While there is cogent evidence that SE supplementation may reduce occurrence of these adverse events as well as decrease the risk of AIT complications and prevent PP hypothyroidism, the current lack of clear cut-off levels for Se administration, which implicates the risk of overdose, at present tends to militate against Se supplementation in this setting [68].

Concerning patients with AIT, the current Guidelines do not recommend Se replacement during pregnancy [69], while there is a lack of randomized control trials (RCTs) to determine the safety and efficacy of Se supplementation in pregnancy at high risk for adverse events, including miscarriage, preeclampsia, preterm delivery, and fetal death. Until such studies are available, AIT patients who are at high risk and have low Se levels, i.e. < 80 μg/l in early pregnancy, or who are carriers of the A allele of the SELENOS1 − 105G/A promoter polymorphism (rs28665122), should be considered for Se supplementation [70]. However, it should be borne in mind that Se has a narrow therapeutic index and the efficacy of treatment follows a U-shape, accompanied by increased side effects above a level of 120 μg/l in serum [70]. Thus, above this level the patient does not need any supplementation. Parallel to Se level measurement, other parameters must also be taken into account before supplementation is started: specifically, thyroid function, dietary iodine and other micronutrient intake, and the subject’s region of residence with regard to its soil Se content. An alternative to Se measurement, in the setting of an individualized approach, is the quantification of serum/plasma SELENOP which is a more stable and sensitive parameter of Se status, albeit reference levels for pregnancy are not well defined.

## Conclusions

The present review is based on a number of studies that have highlighted the importance of adequate Se levels during pregnancy. This particularly concerns at-risk pregnancies, where increasing evidence shows that Se deficiency may be implicated in miscarriage, preeclampsia, retarded fetus intrauterine growth, and preterm birth. Furthermore, inadequate Se may accelerate placental OS and have a negative effect on trophoblast viability when exposed to hypoxia. In addition, pregnant women with AIT in conjunction with low Se levels exhibit an increased risk for adverse pregnancy outcome and postpartum hypothyroidism and therefore require sustained control of AIT. It is hence evident that to enable an optimal pregnancy outcome, physicians should employ an individualized approach, prescribing patient-tailored Se intake within the framework of a balanced diet including, in general, an adequacy of micronutrients, particularly when this concerns expectant mothers at high risk.

## Data Availability

No other source than the references cited.

## Abbreviations

AIT: Autoimmune thyroiditis
CAT: Catalase
DIOs: Deiodinases
DIO1: Deiodinase type 1
DIO2: Deiodinase type 2
DMGDH: Dimethylglycine dehydrogenase
DNA: Deoxyribonucleic acid
DUOX: Dual oxidases
EnGioI: Young Italian Endocrinologists Group - EnGioI
ERK: Extracellular signal-regulated kinase
FT4: Free thyroxine
GSR: Glutathione reductase
GPXs: Glutathione peroxidases
GPX1: Glutathione peroxidase 1
HIV: Human immunodeficiency virus
H2O2: Hydrogen peroxide
H2Se: Selenide
IL-1: Interleukin 1
IL-6: Interleukin 6
INMT: Indolethylamine N-methyltransferase gene
mRNA: Messenger RNA
Met-tRNA: Methionine-translation ribonucleic acid
NADPH: Nicotinamide adenine dinucleotide phosphate
OS: Oxidative stress
O2: Oxygen
02 *: Superoxide radical
PI3K/AKT: Phosphoinositide 3-kinase activation
PP: Postpartum
RCTs: Randomized control trials
ROS: Free radical oxygen species
rT3: Reversing triiodothyronine
Se: Selenium
SeCyS: Selenocysteine
SECISBP2: Selenocysteine insertion sequence (SECIS) binding protein 2 3
SeMet: Selenomethionine
SELENOK: Selenoprotein K
SELENOL: Selenoprotein L
SELENON: Selenoprotein N
SELENOP: Selenoprotein P
SELENOS: Selenoprotein S
sFlt-1: Soluble vascular endothelial growth factor receptor-1
SOD: Superoxide dismutase
T3: Triiodothyronine
T4: Thyroxine
Tg: Thyroglobulin
TGAb: Thyroglobulin antibodies
TH: Thyroid hormone
TNFα: Tumor necrotic factor α
TPOAb: Thyroid peroxidase antibodies
TRXR: Thioredoxin reductases
TSH: Thyroid-stimulating hormone
